# Mining the Volatilomes of Plant-Associated Microbiota for New Biocontrol Solutions

**DOI:** 10.3389/fmicb.2017.01638

**Published:** 2017-08-25

**Authors:** Aurélien Bailly, Laure Weisskopf

**Affiliations:** ^1^Department of Plant and Microbial Biology, University of Zurich Zurich, Switzerland; ^2^Agroscope, Institute for Sustainability Sciences Zurich, Switzerland; ^3^Department of Biology, University of Fribourg Fribourg, Switzerland

**Keywords:** volatile organic compounds, *Pseudomonas*, phytophthora, potato, biocontrol, microbiome

## Abstract

Microbial lifeforms associated with land plants represent a rich source for crop growth- and health-promoting microorganisms and biocontrol agents. Volatile organic compounds (VOCs) produced by the plant microbiota have been demonstrated to elicit plant defenses and inhibit the growth and development of numerous plant pathogens. Therefore, these molecules are prospective alternatives to synthetic pesticides and the determination of their bioactivities against plant threats could contribute to the development of control strategies for sustainable agriculture. In our previous study we investigated the inhibitory impact of volatiles emitted by *Pseudomonas* species isolated from a potato field against the late blight-causing agent *Phytophthora infestans*. Besides the well-documented emission of hydrogen cyanide, other *Pseudomonas* VOCs impeded *P. infestans* mycelial growth and sporangia germination. Current advances in the field support the emerging concept that the microbial volatilome contains unexploited, eco-friendly chemical resources that could help select for efficient biocontrol strategies and lead to a greener chemical disease management in the field.

## Introduction

Since the Neolithic Revolution about 12,000 years ago, the onset of plant domestication and the progressive systematization of agricultural practices have gradually led to monophyletic cropping systems, prone to pathogen outbreaks. Although the modern eras' mechanization, irrigation and chemical field management tremendously increased crop yields, today's agriculture faces the critical dilemma to meet global food demand and preserve environmental resources. In the context of climate change, productivity pressure and societal uncertainty over genetic manipulation, plant diseases and their management increasingly threaten food security and ecosystems. The promotion and intensification of sustainable farming practices relies on new biotechnological developments. Our growing understanding of the benefits brought by plant-associated microbes to crop health and growth has led to the realization that the plant-**microbiome** constitutes an untapped source of potential biocontrol agents, new valuable molecules and farming strategies (Mueller and Sachs, 2015).

KEY CONCEPT 1 **Microbiome and microbiota.**

Often misused as synonyms, these two terms describe distinct definitions of microbial communities. The microbiota denotes the microorganisms that reside in an environmental niche. The microbiome refers to the collective genomes of these microorganisms.

Among the large diversity of microbial secondary metabolites, low molecular-weight **volatile organic compounds** (VOCs) have received growing attention in the past decade. Since the early reports describing the health- and growth-promoting effects of bacterial VOCs on model plants (Ryu et al., 2003, 2004), an increasing number of studies has evidenced the great potential of these gaseous molecules in crop enhancement and protection (reviewed in Bailly and Weisskopf, 2012; Kanchiswamy et al., 2015b). Microbial VOCs (mVOCs) are typically released in a multifarious and dynamic bouquet, essentially originating from the catabolic background, and comprise a majority of low-complexity, rather lipophilic compounds (Schulz and Dickschat, 2007; Blom et al., 2011a; Penuelas et al., 2014; Schenkel et al., 2015). Thus, mVOCs are seen as *bona fide* semiochemicals able to evaporate to the extracellular space, reach target organisms and partition into biological membranes or intracellular compartments. Indeed, microbial emissions have been shown to trigger significant volatile-mediated responses in bacteria (Garbeva et al., 2014; Audrain et al., 2015; Schulz-Bohm et al., 2015; Tyc et al., 2015), fungi (Effmert et al., 2012; Schmidt et al., 2015; Werner et al., 2016), plants (Bailly and Weisskopf, 2012; Pieterse et al., 2014; Kanchiswamy et al., 2015b), and invertebrates (D'alessandro et al., 2013; Davis et al., 2013). Although the molecular mechanisms underlying mVOCs perception by plants remain unclear, numerous studies have demonstrated that this system results in a potent priming of the plant basal immune system, termed **induced systemic resistance** (ISR), conferring broad-spectrum resistance against pathogens. In contrast to pattern-triggered immunity (PTI) and subsequent mounting of SAR, ISR elicitation does not negatively impact growth and productivity; in fact, many ISR-triggering microorganisms were selected for their plant growth-promoting and stress-relieving properties (reviewed in Van Hulten et al., 2006; Choudhary et al., 2007; Yang et al., 2009; Heil, 2010; Huot et al., 2014; Pieterse et al., 2014). Given the origin and chemical properties of mVOCs, these interkingdom cues represent a prospective pool of new functions that need further investigation and development to be delivered to the field (Fernando et al., 2005; Kanchiswamy et al., 2015a,b; Chung et al., 2016).

KEY CONCEPT 2 **Volatile organic compounds.**

VOCs are low-molecular weight, carbon-containing compounds (excluding very simple chemical species, such as carbon monoxide or carbon dioxide) that display high vapor pressure and low boiling point. Biogenic VOCs have been described as *bona fide* semiochemicals in most phyla.

KEY CONCEPT 3 **Induced systemic resistance.**

Induced resistance is a general term describing an induced state of resistance in plants triggered by the local perception of biotic or abiotic cues. Induced systemic resistance (ISR) describes the elicitation of latent plant defenses that systemically protects naive plant parts against future attackers, also termed defense priming. ISR activation depends on jasmonic acid and ethylene hormonal responses and is distinct from the systemic acquired resistance (SAR) engaged by the cellular recognition of microbe-associated molecular patterns (MAMPs), characterized by increased levels of the phytohormone salicylic acid.

Our recent work has focused on late blight, the major worldwide potato disease caused by the oomycete *Phytophthora infestans*. Although this particular pathosystem is obviously distinct from other fungal or bacterial plant diseases, our line of reasoning within this focused review could be extended to a wide range of plant pathogens. Under favorable conditions, *P. infestans* easily spreads from plant to plant through densely planted monocultures and rapidly ravages entire fields (Fry, 2008), and disease forecasting has become a key tool for growers. While conventional field practices control late blight via repeated, preventive applications of broad-spectrum fungicides, organic farming greatly relies on copper-based products toxic to the environment (Dorn et al., 2007; Cooke et al., 2011; Nechwatal and Zellner, 2015). The search for alternative organic solutions using either horticultural extracts, biosurfactants, or applications of plant beneficial bacteria or compounds eliciting plant defenses has not yet yielded reliable market products (Dupuis et al., 2007; Diallo et al., 2011). However, the increase in stringent policies regarding copper release into the environment exerts pressure for the continuation of investigations. *In vitro* work has demonstrated that mVOCs specifically contribute to the inhibition of growth and development of several phytopathogenic fungal or fungal-like genera, including members of *Aspergillus* (Vespermann et al., 2007; Hua et al., 2014; Chaves-Lopez et al., 2015; Gong et al., 2015), *Botrytis* (Huang et al., 2011; Li et al., 2012; Rouissi et al., 2013; Zhang et al., 2013; Parafati et al., 2015), *Fusarium* (Vespermann et al., 2007; Minerdi et al., 2009; Yuan et al., 2012; Tenorio-Salgado et al., 2013; Wang et al., 2013; Cordero et al., 2014), *Penicillium* (Rouissi et al., 2013), *Sclerotinia* (Fiddaman and Rossall, 1993, 1994; Fernando et al., 2005; Vespermann et al., 2007; Giorgio et al., 2015), *Rhizoctonia* (Fiddaman and Rossall, 1993, 1994; Kai et al., 2007; Vespermann et al., 2007; Liu et al., 2008), *Alternaria* (Andersen et al., 1994; Chaurasia et al., 2005; Trivedi et al., 2008; Zhao et al., 2011; Groenhagen et al., 2013), *Pythium* (Chaurasia et al., 2005; Sanchez-Fernandez et al., 2016), and *Phytophthora* (Zhao et al., 2011; Ann, 2012; Sharma et al., 2015).

Our recent study has therefore been centered on the hypothesis that the volatilomes of bacteria naturally associated with potato plants contain active compounds against *P. infestans* and that, once isolated, these antagonists would make ideal candidate biopriming control agents (Hu et al., 2014; Spence et al., 2014; Mahmood et al., 2016).

## Harnessing the plant microbiota volatile metabolome

Throughout their whole lifecycle, land plants are continuously covered by environmental microorganisms colonizing their surfaces, invading intra- and intercellular spaces or building intimate symbiosis. Microbes have evolved life strategies displaying commensal, beneficial, or pathogenic behaviors toward plants to access the metabolic resources they offer. Plants are constantly challenged with biotic cues that need to be processed to balance growth, development and defense programs and achieve optimal fitness (Huot et al., 2014). They have developed a multilayered monitoring strategy that relies on the capacity of each individual cell to perceive molecular effectors and translate them into a systemic signal triggering an alert status in distant organs and on-site defense responses. In addition, recent insights into the host-specific composition of microbial communities have suggested that plants, to some extent, select for their **microbiota** (Bulgarelli et al., 2013; Schlaeppi and Bulgarelli, 2015). The intense competition between microbes for nutrients and favorable niches both at the **rhizosphere** and **phyllosphere** levels might in effect provide a functional addition to the plant immune system, and host-specific microbiomes can essentially be seen as an extension of the plant genome. As laboratory model organisms capable of increasing plant health may prove difficult to transfer to field conditions, the isolation of highly-adapted strains from the plant *in situ* microbiota has a much greater chance of success in antagonist selection processes.

KEY CONCEPT 4 **Rhizosphere and phyllosphere.**

In microbiology, the term rhizosphere refers to the thin volume of soil directly influenced by plant root exudates and root-associated microorganisms, while the phyllosphere describes the above-ground plant surfaces hosting microbial species. Both represent dynamic habitats with drastically different resources and environmental conditions for microorganisms.

We therefore isolated 137 morphologically distinct bacterial strains on different growth media from the rhizosphere and phyllosphere of field-grown potato plants previously infected with *P. infestans* (Hunziker et al., 2015). Subsequent phylogenetic identification of 92 of these strains to the genus or species level using 16S and *rpoD* gene amplicons revealed that *Actinobacteria* and *Proteobacteria* were the most abundant among the isolated organisms. Although our sampling and isolation methods were not exhaustive and higher resolution of OTUs has been described elsewhere (Inceoglu et al., 2011; Barnett et al., 2015), the retrieved strains are *bona fide* potato-associated bacteria. From the 32 bioactive strains pre-selected from a series of dual-culture assays against *B. cinerea, R. solani*, and *P. infestans* growth, *Pseudomonas* species had the highest inhibitory potential.

In order to evaluate the volatile-mediated activity of these strains, we then co-cultured bacterial colonies and five discrete target potato pathogens in physically-separated compartments using the I-plate Petri dish system (Hunziker et al., 2015). This work revealed that (1) *P. infestans* was the most VOC-susceptible target organism, (2) *Pseudomonas* species displayed the highest volatile-mediated activity, and (3) that hydrogen cyanide production could account for a large part of the observed inhibition. The large difference between the susceptibility of *P. infestans* and true fungi to mVOCs could partly be explained by the differing nature of its cell wall. Additionally, in our hands, the increase in VOCs-mediated inhibition of fungal and fungal-like species' radial growth seemed to correlate with slower growth speed (Groenhagen et al., 2013; De Vrieze et al., 2015; Hunziker et al., 2015).

The contribution of volatile HCN to the biocontrol properties of *Pseudomonas* strains against fungal pathogens has been known for 20 years, since the demonstration of the suppression of *Thielaviopsis*-induced tobacco black root rot by the cyanogenic *P. protegens* CHA0 but not by its isogenic mutant *P. protegens* CHA77 (Voisard et al., 1989; Haas and Defago, 2005; Rudrappa et al., 2008; Lanteigne et al., 2012). In the same extent, other inorganics of bacterial origin, such as ammonia, or hydrogen sulfide are suspected to account for a significant part of the target organism growth inhibition (Bernier et al., 2011; Shatalin et al., 2011; Weise et al., 2013). However, throughout our experimental work, no correlation was found between *P. infestans* mycelial growth and bacterial NH_3_ production (Hunziker et al., 2015). Moreover, the oomycete was still significantly inhibited when exposed to the volatile blend of the cyanide-deficient mutant CHA77, thus indicating that beside HCN and NH_3_, *Pseudomonas* strains release other potent volatiles against *P. infestans*.

This indicated that the identification and quantification of the volatile chemical species composing the natural emissions of cyanogenic and non-cyanogenic bacteria is a prerequisite to the evaluation of their contribution to the inhibitory impact on the target pathogen (Kai et al., 2007, 2009; Campos et al., 2010; Effmert et al., 2012). Such approaches, essentially based on molecule-trapping techniques and gas chromatography-mass spectrometry (GC-MS) platforms, became the standard in the field (Schulz and Dickschat, 2007), thus generating vast amounts of data in which non-abundant and/or non-readily available chemical species are generally overlooked. Indeed, while a large body of literature has reported the inhibitory activity of a broad range of bacterial volatilomes against several discrete fungal or fungal-like pathogens, the identity of single active VOCs remains elusive. In many studies, the application of identified compounds as physiologically relevant amounts of synthetic molecules rarely reached the inhibitory effects observed with natural VOC bouquets, suggesting that volatile blends act in a multifactorial manner (Yuan et al., 2012; Groenhagen et al., 2013; Chaves-Lopez et al., 2015). Recent studies have tentatively reconstituted artificial mixtures of several prominent volatile species and reported their greater effects when compared to single compound applications, suggesting that volatiles interact synergistically (Cortes-Barco et al., 2010a; Fialho et al., 2010, 2011a,b; Mitchell et al., 2010; Naznin et al., 2013; Riyaz-Ul-Hassan et al., 2013).

**Figure 1:**
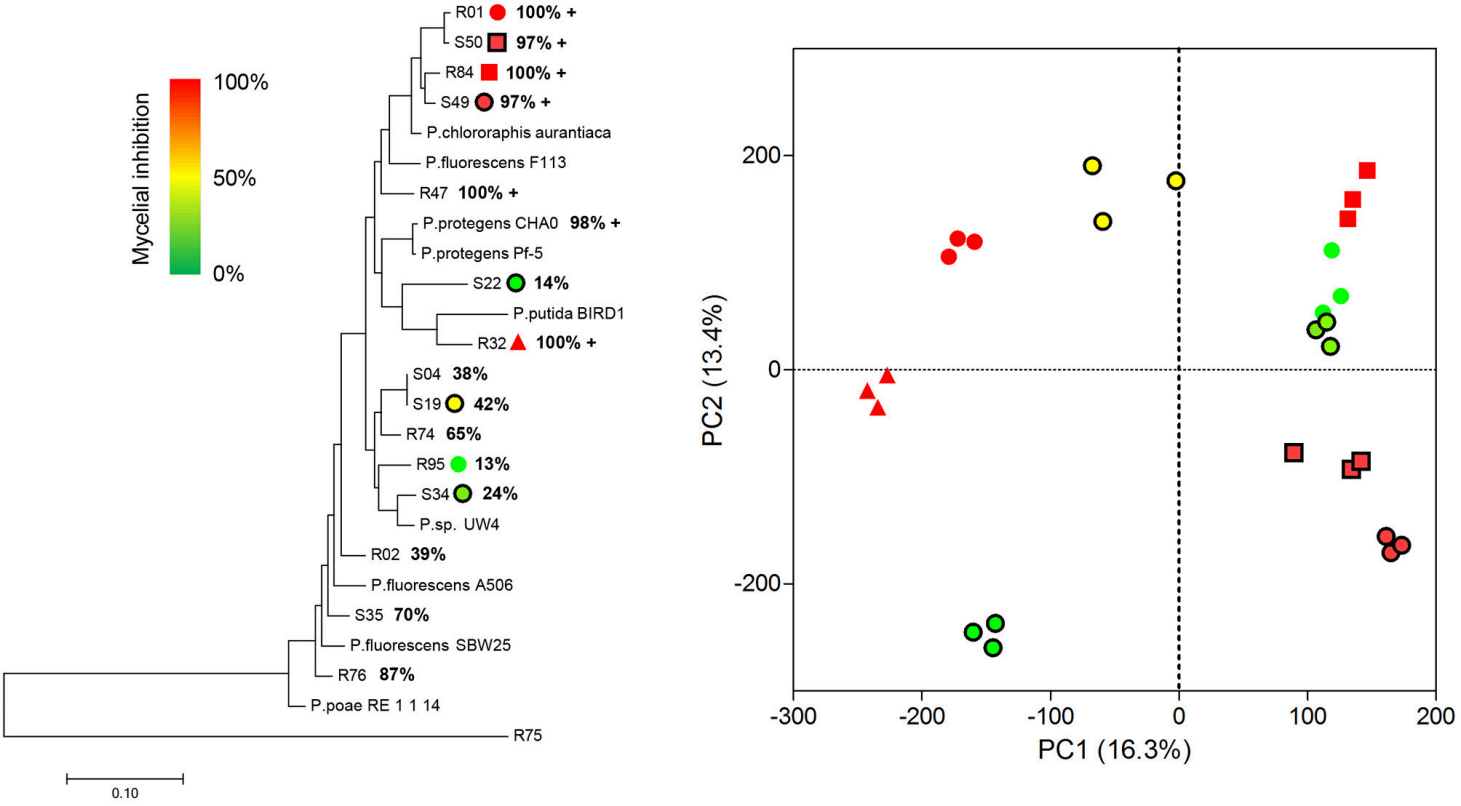
Volatile organic compounds production in *Pseudomonas* sp. does not mirror phylogenetic relationships. **Left**, maximum-likelihood-based phylogenetic tree of 24 *Pseudomonas* sp. calculated from multi-locus sequence alignments of 16s rRNA, gyrB, rpoD, and rpoB genes concatenations using the MEGA software. Bars indicate mean average base substitutions between sequences. Note that the *Flavobacterium* sp. R75 stands as an outlier. Bold percentage values represent the volatile-mediated inhibition of *P. infestans* mycelial growth for each tested strain. R and S refer to strains from rhizospheric and phyllospheric origin, respectively; +, cyanogenic stain. **Right**, 2-D scores plot between the highest PC scores from a principal components analysis of the total ion chromatograms of 9 selected *Pseudomonas* isolates. Individual biological replicates of each strain are identified according to the legend in the tree **(left)**; each data point is color-coded according to the VOCs-mediated *P. infestans* mycelial growth inhibition exerted by the corresponding strain. Spectral data were processed using the MetaboAnalyst 3.0 software following this procedure: detection of Gaussian-fitted peaks (4 s fwhm, binning, integrated area of original peak), followed by alignment and grouping according to their masses and retention time after retention time correction. Data were subsequently filtered based on interquantile range, then normalized by the sample median and finally generalized log-transformed. Data scaling and outlier removal were left in automatic mode. Explained variances are shown in brackets.

**Figure 2:**
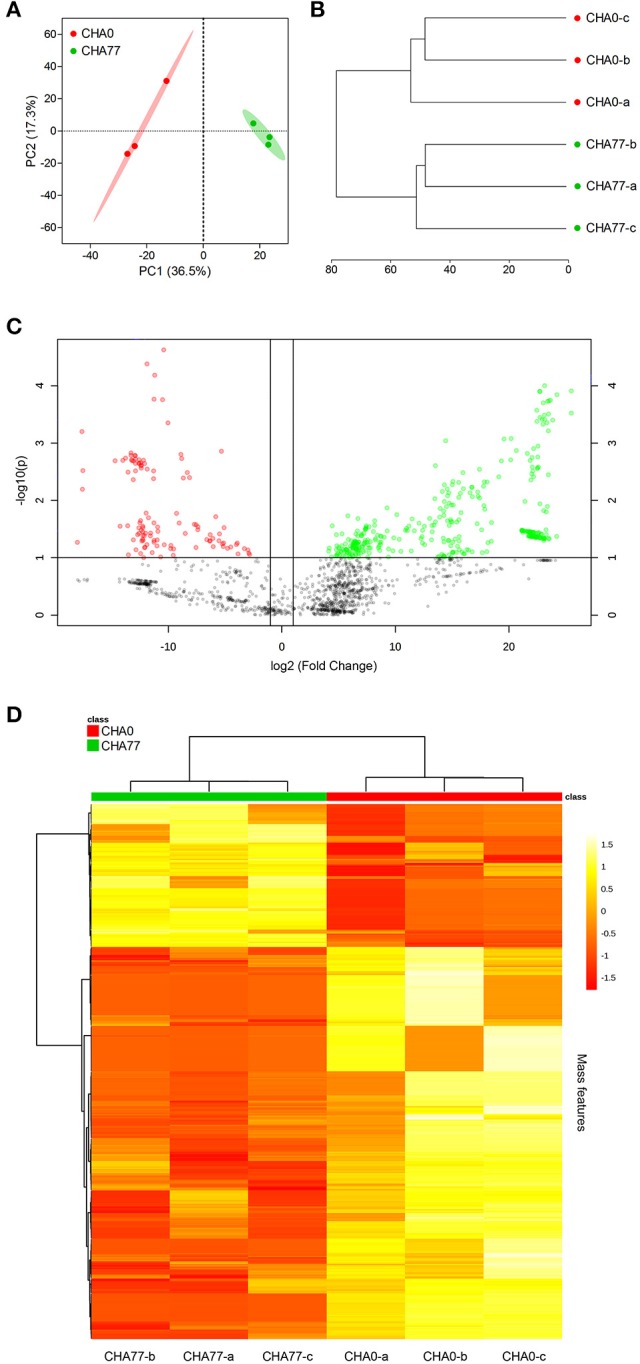
*Pseudomonas protegens* CHA0 and its isogenic, cyanide-deficient mutant CHA77 express distinct volatilomes. **(A)** PCA 2-D scores plot from total ion chromatograms processed as in Figure 1. Shaded areas represent the 95% confidence interval; explained variances are shown in brackets. **(B)** Hierarchical clustering dendrogram of the biological replicate volatilomes (Euclidean distance similarity and Ward's linkage clustering). **(C)** Volcano plot of mass features differentially detected in CHA77 compared to CHA0 volatilomes selected by fold change (threshold = 2) and *t*-test values (threshold = 0.1). Note the higher number of mass features with positive fold change (green) compared to negative fold change (red) in the HCN-deficient mutant. **(D)** Heat map and hierarchical clustering of the top 300 *t*-test selected mass features. Spectral data were processed as described for Figure 1.

**Figure 3:**
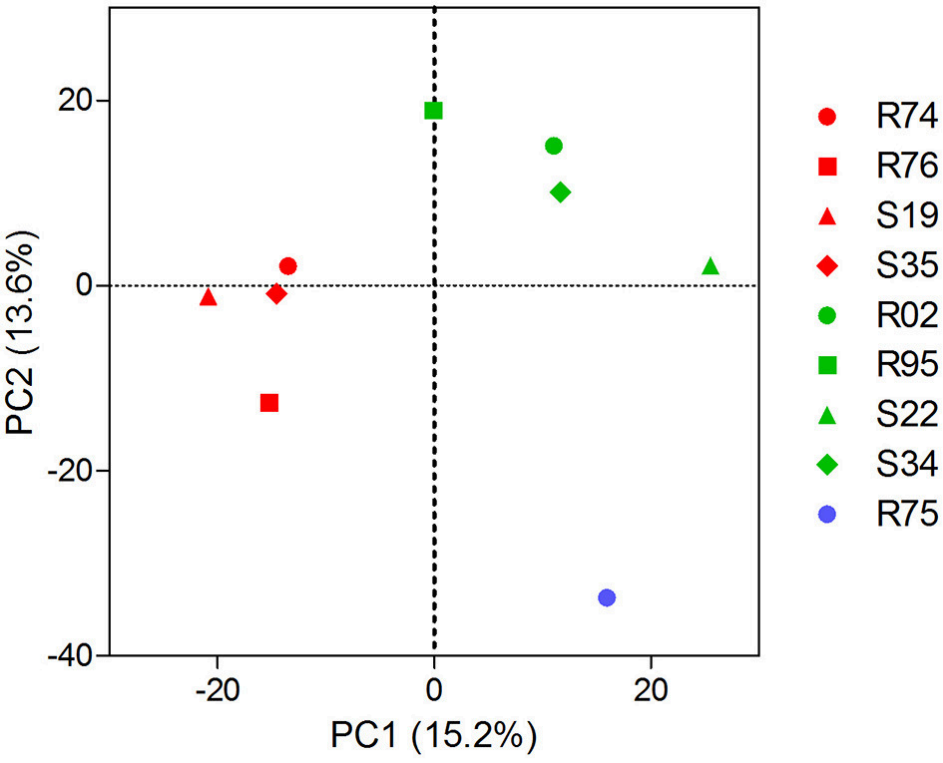
Non-cyanogenic *Pseudomonas* sp. chemoprofiles may contain compounds explaining the different inhibition potential against *P. infestans*. 2-D scores plot between the highest PC scores from a principal components analysis of the total ions chromatograms of 9 non-cyanogenic strains processed as in Figure 1. Data points represent the centroids of 3 biological replicates. Note that, besides a net separation between high-activity (red) and low-activity strains (green), the variance explained by each component is low. The chemoprofiles of the non-*Pseudomonas* strain R75 (blue) separate from *Pseudomonas* chemoprofiles. Explained variances are shown in brackets. Spectral data were processed as described for Figure 1.

Extending our initial investigation of 8 *Pseudomonas* volatilomes (Hunziker et al., 2015), we collected and identified the compounds emitted by CHA0, CHA77, and 16 of our selected *Pseudomonas* strains grown on lysogeny broth plates for 24 h, under conditions mimicking our I-plate assays. We hypothesized that each strain's specific volatile-mediated inhibition potential could be explained by either a different population or different amounts of single VOCs in the volatile blends. The obtained chemoprofiles comprised volatile motifs previously identified in *Pseudomonas* biogenic emissions, with 1-undecene and dimethyl disulfide (DMDS) being the most prominent species (Lemfack et al., 2014; Hunziker et al., 2015), and appeared relatively conserved, thus supporting the concept that volatile signatures could help discriminate microbial genera or species (Thorn et al., 2011; Shestivska et al., 2015; Dryahina et al., 2016; Neerincx et al., 2016). However, detailed comparisons of the collected GC-MS data failed to identify the chemical features responsible for the strains VOCs-mediated inhibitory effects (Figure 1). The genetic proximity of our *Pseudomonas* isolates did not necessarily translate into identical chemoprofiles (Shestivska et al., 2012; De Vrieze et al., 2015), and it appeared that the impact of the rhizospheric or phyllospheric origin was negligible in our sampling. A previous study investigating the effects of volatiles emitted by closely-related *Burkholderia ambifaria* strains with discrete isolation origins on various target organisms also reported very similar, yet different VOC chemoprofiles, leading to very subtle changes in the targets' responses (Groenhagen et al., 2013). It is highly plausible that the rich LB medium on which we grew our strains during headspace collection did not select for and reflect the particular metabolic potential of our test-strains, but we expect the collected spectral data to mirror our inhibition assays. Moreover, univariate pair fold change analysis between CHA0 and CHA77 chemoprofiles displayed over 90 significantly different mass features (*t*-test, *p* < 0.005), including enrichments in dimethyl trisulfide (DMTS), s-methyl methanethiosulfonate (MMTS) and aminoacetophenone production in the non-cyanogenic mutant (Figure 2). Interestingly, HCN itself is not detected in standard GC-MS methods and so does not impact the differences observed between mutant and wild-type strains' chemoprofiles, thus a change in the synthesis of one particular volatile can lead to a drastic alteration of the overall volatile profiles emitted by otherwise isogenic strains. When focusing on non-cyanogenic isolates, relatively poor PCA clusterings of the total ion GC-MS chromatograms tended to separate the most active strains' chemoprofiles from low activity ones (Figure 3), although no single compound or chemical pattern seemed to unequivocally explain the blend's effect. The abundance or the detection frequency of compounds that do not substantially contribute to the total effect of the whole volatile blend may impede the description of bioactive patterns. Thus, in order to strengthen chemoprofiling data and identify key chemical species, the precise determination of the inhibition potential of individual substances is essential.

## A pharmacological approach to exploring volatile potential

**Figure 4:**
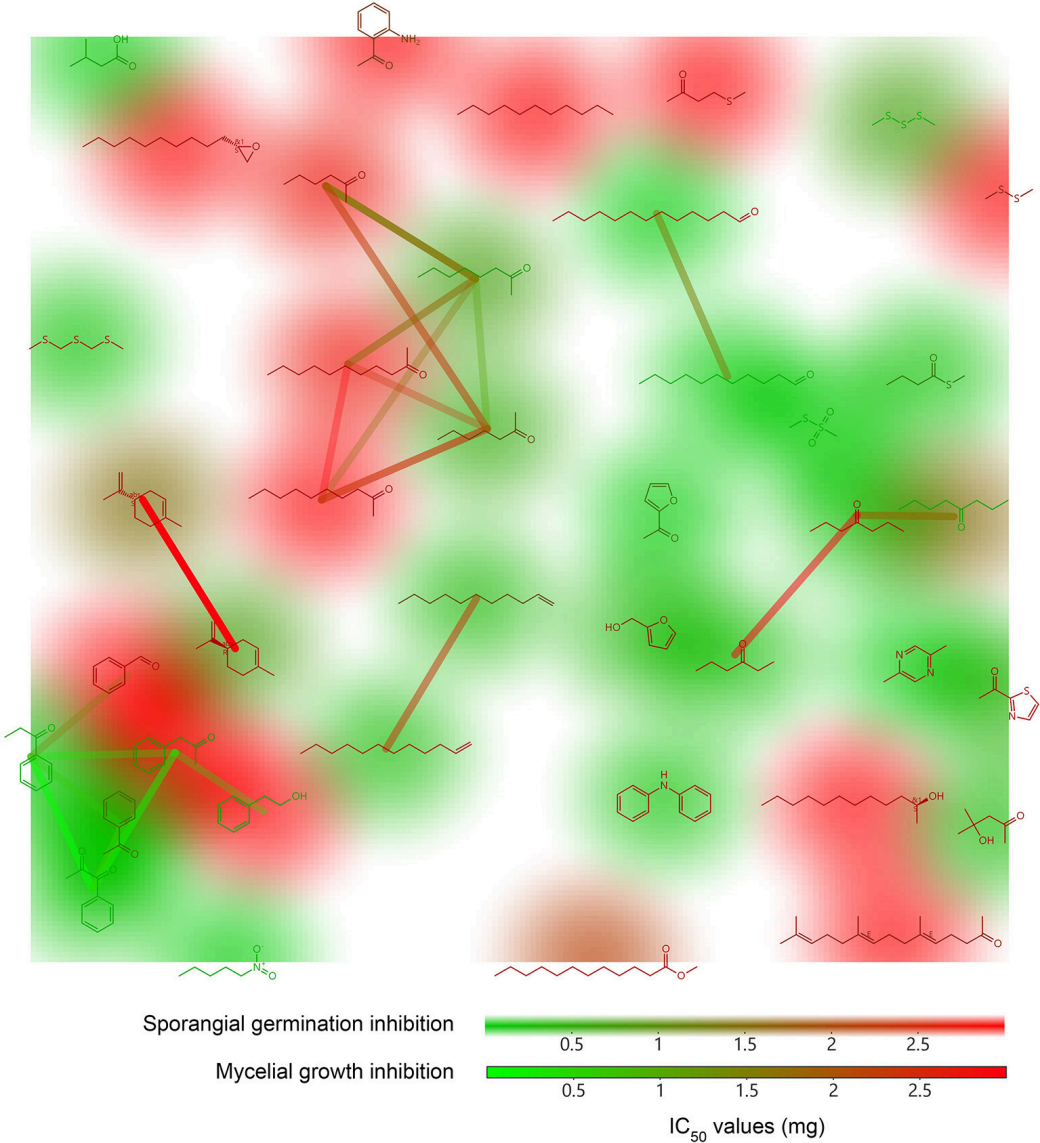
Structure-activity landscape plots of 40 *Pseudomonas* volatile organic compounds tested against *P. infestans* Neighbor analysis, Murcko scaffold analysis and FragFp structure similarity analysis were performed using the Actelion Pharmaceuticals DataWarrior 4.4.3 software. Structures are colored according to their *P. infestans* mycelial growth inhibitory activity while structure backgrounds display sporangia germination inhibition. Connection lines between different structures indicate the number of neighbor molecules (red, low number; green, high number).

The large amount of data contained in mass spectra, coupled to the difficulties in identification of the chemical structures they refer to, make systematic testing of the bioactivities of individual compounds a daunting task. Moreover, the limited number of comparative studies involving different microbial genera (Kai et al., 2009; Blom et al., 2011a,b; Berrada et al., 2012) does not allow the assessment of candidate active volatiles that may be present or absent in the respective volatilomes, leaving a striking knowledge gap. With the aim of assigning a weight function to our chemoprofiles, we attempted to characterize the precise contribution of 40 commercially available pure substances identified from the natural emissions of our isolates by assessing their biological activity against several stages of *P. infestans* life cycle. Although non-exhaustive, this series of assays revealed that a majority of *Pseudomonas* volatiles possess low to mild inhibitory power against *Phytophthora* and probably act synergistically on the target organism (De Vrieze et al., 2015). Although limited to a small panel of simple compounds, the quantitative relative IC50 values derived from dose-dependent *P. infestans* mycelial growth and sporangial germination inhibition assay allow for basic structure-activity relationship exploration (Figure 4). First, *P. infestans* sporangia appeared more sensitive to mVOCs exposure than mycelia, especially to aliphatic compounds, such as long chain aldehydes (undecanal and tridecanal), alkenes (1-undecene and 1-dodecene), and short-chained ketones (2-octanone, 2-heptanone, 4-heptanone, 3-hexanone, and 4-hydroxy-4-methyl-2-pentanone), while 2-dodecanol or undecane were found to be inactive. Interestingly, similar activities have been previously reported against *Alternaria alternata* germ tube growth for this chemical family (Andersen et al., 1994), implying that these lipoxygenase products may cause broad-spectrum interference to fungal and fungal-like germ tube development. This is further supported by the fact that the exposure to a subset of ketones, such as 3-hexanone triggered severe malformations in *P. infestans* germ tubes (De Vrieze et al., 2015). The closely-related compounds furfuryl alcohol and acetyl furan, as well as three of the six phenyl-ketones tested also performed well against *P. infestans* germination. Acetophenone derivatives are well-described antifungals thought to target the fungal cell wall (Soberon et al., 2015; De Aguiar et al., 2016). A high level of inhibition also resulted from exposure to diphenylamine and 2,5-dimethylpyrazine treatments; however we cautiously consider these compounds as artifacts originating from the medium. Secondly, both mycelia and sporangia showed high sensitivity to sulfur-containing compounds, such as bis(methylthiomethyl)sulfide, s-methylbunathioate, MMTS and DMTS, isovaleric acid and nitropentane. Nitroalkanes are renowned toxic substances for animals, but to the best of our knowledge, no particular study has investigated their effect on fungal growth. However, some very potent non-volatile antimicrobials display an active nitro-group, for instance nitrofurazone, metronidazole and chloramphenicol. DMDS and to a lesser extent DMTS and MMTS have been repeatedly shown to exert broad-range antifungal activities, probably via their capacity to reduce protein sulfhydryl groups and readily oxidize into highly reactive sulfur-acids, and are considered as prominent antimicrobials in the *Brassicacea* and *Allioideae* (Fernando et al., 2005; Kocic-Tanackov et al., 2012; Groenhagen et al., 2013; Zhou et al., 2014). DMDS-containing products are already marketed as soil fumigants for the suppression of soil-borne plant diseases. However, DMDS poorly performed in our assays, with the exception of zoospore activity. The inhibition of *P. infestans* radial growth and sporangia germination by isovaleric acid is especially interesting as this compound was shown to trigger the germination of *Agaricus bisporus* spores (Rast and Stauble, 1970) but not of ectomycorrhizal fungi (Fries, 1978), and to inhibit *Fusarium* growth (Monnet et al., 1988). Most other free fatty acids are considered as broad-range fungal inhibitors interfering with membrane composition (Pohl et al., 2011).

Finally, few compounds were solely active against *P. infestans* mycelia without also impacting sporangia development and function, and these belong to the chemical groups discussed above. Furthermore, these molecules, namely 2-phenylethanol, 2-phenylacetone, 2-octanone and 4-octanone, showed mild-to-low inhibitory power. Remarkably, several reports focusing on the antifungal activity of ketones identified from *Bacillus* species (Fernando et al., 2005; Arrebola et al., 2010; Yuan et al., 2012; Zhang et al., 2013) concluded that long-chain ketones, such as 2-nonanone and 2-decanone demonstrated strong inhibition activity against fungal species. However, in our work, long-chain ketones treatments did not provide satisfactory inhibition of *P. infestans*, although 2-undecanone exposure led to a strong densification of the mycelial mat (De Vrieze et al., 2015). Similarly, a study by Chaves-Lopez et al. (2015) focusing on single volatiles from *Bacillus* documented that only short-chain ketones like 2-butanone were efficient against *Fusarium oxysporum* and *Moniliophthora perniciosa* growth. These discrepancies may simply be explained by the different sensitivities of the studied target organisms to mVOCs, but they may also find their source in the low pharmacological resolution of the employed methodology. In Yuan et al. (2012), *F. oxysporum* was exposed to 200 μl of a subset of *Bacillus* VOCs while in Chaves-Lopez et al. (2015), 25 and 50 μl of another subset of *Bacillus* VOCs were applied. Regardless of the boiling point of the particular compounds, these amounts represent tens to hundreds of milligrams introduced into the headspace, far beyond the actual production capacity of the bacterial cultures (De Vrieze et al., 2015; Shestivska et al., 2015). As VOCs readily diffuse to the environment, attention should be paid to substances with the highest potency and low dose efficacy. We therefore advocate the systematic assignment of pharmacological values based on standardized bioassays against the investigated target organisms, to the chemical species identified from microbial volatilomes. Our current work strives for the successful implementation of these valuable data layers that will allow deeper assessment of the ecological impact of biogenic microbial emissions and greatly help in pinpointing potent molecules or cornering active chemical backbones produced by various bacterial genera. Alternatively, these compounds could provide leads to drug discovery strategies, as exemplified by volatile benzothiazole (Herrera Cano et al., 2015; Zhao et al., 2016) and 2,4-diacetylphloroglucinol (Lanteigne et al., 2012), or help to select for the most appropriate antagonists from a panel of bioactive mVOCs.

## Toward the development of a volatilomics platform for plant-microbes interactions

As an emanation of the metabolome of a given organism in a given condition, the collected volatile blends represent only snapshots of a more complex phenomenon. Different substrate use, various growth conditions and genetic mutations are just some of the factors that directly influence the chemical composition of volatilomes (Fiddaman and Rossall, 1994; Blom et al., 2011a; De Vrieze et al., 2015). Furthermore, the natural conditions and environmental cues leading to the production of particular volatile species or signatures have not yet been resolved, and their biological relevance in biocontrol contexts remain to be assessed outside *in vitro* systems (issues reviewed in Schmidt et al., 2015; Chung et al., 2016). Therefore, the definition of the **volatilome** is not fixed to the capacity to enzymatically produce a particular compound as engraved in the genomes, but is instead relative to the dynamics of headspace compound release. In analogy to transcriptional patterns, the effect of mVOC emissions on a given target organism may depend on the production of a combination of key chemical species. Yet, in order to better characterize the impact and functions of mVOCs in interspecies relationships, a transition from low-scale individual studies to global data mining platforms is required. The experience gained in other -omics fields, especially the emergence of transcriptomic data, has led to the organization of public data repositories and the creation of resourceful toolsets that have tremendously stimulated research over the last 15 years, such as the NCBI Gene Expression Omnibus (Edgar et al., 2002). However, to a large extent, the sum of complex chemical information related to volatile production by microorganisms gathered in laboratories scattered around the world remains underexploited. As no centralized platforms exist that would allow comparative, statistically-driven exploration of published datasets, the era of metadata analysis of volatilomes has been delayed. The standardized procedures instigated in breath research (King et al., 2011; De Lacy Costello et al., 2014; Broza et al., 2015) should inspire investigators interested in the volatilomes of plant-associated microbiota. Recent attempts to pull together and unify data issued from the literature has resulted in the mVOCs (http://bioinformatics.charite.de/mvoc/; Lemfack et al., 2014) or the KNApSAcK Metabolite Ecology (http://kanaya.naist.jp/KNApSAcK/; Abdullah et al., 2015) databases, yet such initiatives require further development to become valuable instruments. Ideally, standardized NMR/MS peak lists or LC/GC-MS spectra (converted into exchange formats, such as NetCDF or mzXML) obtained from biological replicates would populate a growing database of discrete organisms, strains and experimental conditions that could serve as a basis for exploratory statistical analyses using existing metabolomics tool suites, such as MetaboAnalyst (Xia et al., 2015) or XCMS (Smith et al., 2006). Such advances would help to fill critical knowledge gaps, i.e., the determination of a core volatilome in a given species, the co-occurrence of underrepresented low-abundance mVOCs and the actual composition of emissions released by microorganisms growing in the rhizosphere or phyllosphere. Taken together, this information will provide key concepts to convert the explorative academic knowledge into concrete crop disease control solutions.

KEY CONCEPT 5 **Volatilome.**

The volatilome, also referred to as volatolome, defines the sum of volatile or semi-volatile organic compounds emitted by a biological system under specific experimental conditions. As the transcriptome describes the dynamic expression of genes through mRNA level detection, the quantitative identification of chemical species in the volatilome reflects the dynamic metabolic activity of the studied organism.

## The challenging transition to the field

The concept of exploiting microbial populations hosted by plants to benefit crop health against one or more plant pathogens and productivity is ancient, but has received increasing attention in the past decades, especially in view of the potential biological and ecological functions conferred by rhizospheric and phyllospheric bacterial species (Zahir et al., 2003; Choudhary et al., 2011; Kim et al., 2011; Kumar et al., 2016). Biocontrol strains can bestow disease suppression via competition or parasitism against the targeted pathogens, antibiotic production, cell wall degradation or plant ISR elicitation. The most effective antagonists should display a range of microbicidal properties, as illustrated by *Pseudomonas* species potent in the production of a variety of phenazines, DAPG, pyrollnitrin, HCN (Lanteigne et al., 2012; Loper et al., 2012), and in our opinion, novel efficient mVOCs. These potentials are encoded in the genomes of the microbes and therefore, ever-decreasing DNA sequencing costs allow the prospective mining of genomes for desired functions (Loper et al., 2012).

There has been remarkable progress in defining biocontrol agents and their spread to the environment (Bale et al., 2008) which raises hopes for operational, intensive and yet sustainable agriculture in the next decades. However, regardless of the efforts made toward intensification of bioprospecting, the current bottleneck in delivering tangible applications to farmers results from difficulties in producing formulations suitable for modern agriculture (Lucy et al., 2004; Choudhary et al., 2011; Pérez-Montaño et al., 2014; Velivelli et al., 2014). The potency of biological agents and of (soluble) microbial derivatives has already been well-documented, and had grown into agronomical products (e.g., Mycostop® and Rhizoplus®, utilizing *Bacillus* species; Biocon® and Ecofit®, with *Trichoderma sp*. as active ingredient, or Cerall® and Proradix® containing *Pseudomonas* sp.), but the development of VOC-derived technologies is still embryonic. However, the successful volatile-based mating disruption of pest insects semiochemicals (Reddy and Guerrero, 2010; Lance et al., 2016) stands for an encouraging proof-of-concept. The rather sharp transition from the laboratory to the field has often been smoothed by a switch from model plants like *Arabidopsis thaliana* to economically important plants and greenhouse experiments. The volatile compound 2,3-butanediol, well-studied in *Arabidopsis* (Ryu et al., 2003, 2004; Farag et al., 2006; Han et al., 2006; Cho et al., 2008; Cortes-Barco et al., 2010a,b), was reported to reduce *Colletotrichum*-mediated anthracnose symptoms in *Nicotiana benthamiana* (Cortes-Barco et al., 2010b) and to protect *Agrostis stolonifera* against the fungal pathogens *Microdochium nivale, Rhizoctonia solani*, or *Sclerotinia homoeocarpa* (Cortes-Barco et al., 2010a). Still, in a recent field trial, attempts to reproduce *in vitro* results obtained with 3-pentanol and 2-butanone showed limited protection against a pathogenic *Pseudomonas syringae* (Song and Ryu, 2013), thus underlining the difficulties in delivering mVOC-based technology. In the case of our potato-*Phytophthora* pathosystem, we verified that the isolated *Pseudomonas* strains did not compromise plant health or growth in greenhouse pot cultures. The inoculated potato cultivars did not display any phytotoxicity symptoms or growth defects; but neither strain treatments resulted in growth enhancement (Guyer et al., 2015 and unpublished results). In addition, the ability of our candidate bioncontrol strains to colonize roots and survive on the potato phylloplane was assessed after sprout inoculation or leaf spraying, respectively. The large majority of the isolates demonstrated good rhizocompetence and successfully colonized plant shoots, both in the greenhouse and the field conditions (Guyer et al., 2015). As microbial competition for nutrients and ecological niches on the plant surfaces certainly contributes to the antagonistic activity of competent bacterial strains (Innerebner et al., 2011; Ghirardi et al., 2012; Vorholt, 2012), isolates naturally associated with potato plants have the highest chance to be artificially reintroduced to a crop for control purposes. The promising protective effects measured in dual culture assays and leaf disc infection experiments however, have not yet been transposed to successful field trials (Guyer et al., 2015).

The direct contribution of microbial VOCs in disease suppression in the open field remains elusive, and a study by Sharifi and Ryu (2016) argues that the VOCs-mediated elicitation of ISR is the primary factor in warding off pathogens, while direct inhibition via volatiles has only a minor impact. However, recent investigations by Tahir and colleagues demonstrated that VOCs emitted by well-studied suppressive *Bacillus* species act at multiple levels against the tobacco wilt agent *Ralstonia solanacearum*. Indeed, while *in vitro* work showed that exposure to the *Bacillu*s volatile compounds decreased *Ralstonia* growth and viability and led to substantial defects in cell integrity and mobility, they as well triggered major changes in the expression of *Ralstonia* genes fundamental to disease progression (Tahir et al., 2017). Furthermore, tobacco plants exposed to *Bacillus* emissions and pure identified VOCs increased their transcription levels in key defense-related genes, such as *NPR1* and *EDS1*, thus engaging systemic resistance and resulting in suppression (Tahir et al., 2017). It is therefore conceivable that bacterial volatiles contribute both directly and indirectly to the observed biocontrol properties of *Bacillus*, and that bacterial VOCs bouquets generally act as multifactorial, sequential or simultaneous signals on both pathogens and hosts.

The argument that volatiles dissipate in the environment and never reach efficient inhibiting doses may be valid at a macroscopic scale; nevertheless, competition between microbes on plant surfaces occurs in matrixes like the root mucilage or closed compartments, such as the sub-stomatal chamber, where well-adapted bacterial species may prosper and accumulate higher levels of VOCs. As these environments represent favored entry points for pathogens, we believe that the volatilome forms part of the bacterial arsenal and provides a supplementary line of plant defense. Future disease management integrating the use of biological agents for their water-soluble and volatile features in decision-making processes will lead to alternative solutions to effectively reduce pesticide and fertilizer use in an economically and environmentally sound manner.

## Author contributions

AB and LW wrote the manuscript.

### Conflict of interest statement

The authors declare that the research was conducted in the absence of any commercial or financial relationships that could be construed as a potential conflict of interest.
